# Convergent Metabolic Pathways in MASH Therapeutics: An AMPK‐Centric Analysis

**DOI:** 10.1111/jcmm.71023

**Published:** 2026-01-16

**Authors:** Seungchan Choi, Jin‐Seok Jung, Yie‐sung Seo, Sungmin Song, Jeehye Ham, Hannah Chung, Yousef Ramadan, Kangchan Choi

**Affiliations:** ^1^ Regeneration Medicine Research Center Yonsei University Wonju College of Medicine Wonju Republic of Korea; ^2^ Department of Biomedical Laboratory Science Yonsei University Wonju Republic of Korea; ^3^ School of Medicine Trinity Medical Sciences University Kingstown Saint Vincent and the Grenadines; ^4^ School of Medicine Trinity Medical Sciences University Warner Robins Georgia USA

**Keywords:** AMP‐activated protein kinase, GLP‐1 receptor agonist, MASH, MASLD, resmetirom, SGLT2 inhibitor, statins

## Abstract

Metabolic dysfunction‐associated steatohepatitis (MASH) is a leading cause of liver‐related morbidity driven by systemic metabolic dysregulation. The recent approval of resmetirom and the clinical success of GLP‐1 receptor agonists have heralded a new era in MASH therapy, yet a convergent understanding of the complex mechanisms of these diverse agents is lacking. This review proposes a mechanistic framework centred on the convergent signalling of AMP‐activated protein kinase (AMPK), a master regulator of hepatic energy homeostasis. We examine key metabolism‐based therapeutics—pioglitazone, GLP‐1 receptor agonists, SGLT2 inhibitors, resmetirom and statins—to delineate how distinct upstream triggers converge on AMPK. Synthesising the latest evidence, we clearly delineate how each drug class activates AMPK either indirectly—through systemic effects like weight loss and glycemic control—or via direct actions on hepatocytes. We specifically contrast the liver‐targeted action of resmetirom with the predominantly systemic effects of semaglutide and discuss the ‘epigenetic lock‐in’ hypothesis, wherein chronic metabolic stress perpetuates the disease state. Based on this framework, we propose rational strategies for combination therapy. In conclusion, this AMPK‐centric framework provides a novel lens for understanding the complex pharmacology of MASH drugs and offers a valuable clinical roadmap for personalising treatment strategies to individual patient phenotypes.

## 1 Introduction

Metabolic dysfunction‐associated steatotic liver disease (MASLD) and its progressive variant, MASH (formerly NASH), have been redefined to highlight the critical significance of metabolic dysfunction in fatty liver disease [1]. MASH is marked by hepatic steatosis accompanied by inflammation and hepatocellular damage, potentially advancing to fibrosis, cirrhosis and hepatocellular carcinoma [2, 3]. Due to the worldwide epidemics of obesity and type 2 diabetes, MASH has emerged as a primary contributor to liver‐related morbidity [4]. Until the recent approval of resmetirom, no drug was specifically labelled for MASH, leaving lifestyle modification as the standard of care [5]. However, pioglitazone, a thiazolidinedione targeting insulin resistance, has long been recognised as an effective off‐label therapy [6]. Supported by Level 1 evidence from landmark trials, it is recommended by major guidelines (such as AASLD) for biopsy‐proven MASH, particularly in patients with type 2 diabetes [7]. Building on this foundation, new discoveries are further transforming the therapeutic landscape. The FDA gave resmetirom (brand name Rezdiffra) accelerated approval in March 2024 [8, 9]. It is the first drug for noncirrhotic MASH with fibrosis [10]. It is a selective thyroid hormone receptor‐β agonist. A Phase 3 trial of semaglutide (a GLP‐1 receptor agonist) also showed significant histological improvements, with 62.9% of patients achieving NASH resolution (corresponding to MASH resolution) and a notable fibrosis improvement rate [11]. Such results set the stage for regulatory review. These changes mark the beginning of a new era of MASH therapies that target metabolism.

Even with these improvements, there are still many mechanistic questions. The pathogenesis of MASH is closely associated with systemic metabolic factors, including insulin resistance, elevated fatty acids and pro‐inflammatory signals [12]. Such evidence indicates that therapeutic interventions must facilitate enhancements in both hepatic and systemic levels. A common thread among new treatments is the activation of energy‐sensing pathways, especially AMP‐activated protein kinase (AMPK), which helps the liver break down fatty acids, stops lipogenesis and reduces inflammation and fibrosis [13]. Simultaneously, there is an increasing acknowledgment of the ‘epigenetic lock‐in’ phenomenon: metabolic stress may trigger epigenetic alterations in hepatic cells (e.g., DNA methylation, histone modifications) that sustain the disease even when metabolic parameters enhance. These observations may elucidate the reasons behind patient relapse following weight reduction or the persistent advancement of fibrosis despite the resolution of steatohepatitis [14, 15, 16]. Consequently, genuine and enduring therapeutic success may necessitate the disruption of the epigenetic cycle alongside the rectification of metabolic dysfunction.

This review offers a comprehensive examination of five principal therapeutic modalities—pioglitazone, SGLT2 inhibitors, GLP‐1 receptor agonists, resmetirom and statins—within a convergent mechanistic framework (Table 1). We incorporate the most recent evidence (up to late 2025) to elucidate the mechanisms by which each class operates, respond to contentious issues highlighted in recent critiques (including the direct hepatic actions of GLP‐1 receptor agonists [28] and the hepatic versus systemic effects of SGLT2 inhibitors [29]), and suggest how these findings can be utilised in combination and personalised therapy.

**TABLE 1:** Mechanistic claims and evidence quality for metabolism‐based MASH therapeutics.

Therapeutic class	Mechanistic claim (AMPK‐Associated)	Primary evidence source	Dominant downstream effects	Evidence strength
Pioglitazone	PPARγ‐Adiponectin‐AMPK axis activation	Human RCTs Mech. Studies	DNL ↑ FAO ↑ Insulin Sensitivity ↑	High [17, 18, 19]
SGLT2 Inhibitors	Energy deficit → LKB1‐AMPK activation^a^	Rodent Models (High consistency)	Autophagy ↑ Inflammation ↓ eNOS/NO ↑	Moderate [20, 21]
	Reduced HIF‐1⍺ Improved endothelial function	Rodent Models Human Ex Vivo		
GLP‐1 RAs	Indirect AMPK activation via weight loss	Human RCTs (Systemic effects)	Lipotoxicity ↓ Inflammation ↓	High [11]
	Direct hepatocyte AMPK activation^a^	Rodent Models & In vitro (Controversial)		Low [22]
Resmetirom	THR‐β mediated mitochondrial oxidation	Human Trials (Biomarker supported)	DNL ↓ FAO ↑ Mithochondrial Health ↑	High [23, 24]
	Direct AMPK phosphorylation	Rodent Models		Moderate [25]
Statins	Inhibition of RhoA/ROCK signalling	Rodent models & In vitro	RhoA/ROCK ↓ HIF‐1⍺ ↓ Endothelial Function ↑	High [26]
	LKB1‐AMPK activation via cellular stress			Moderate [27]

*Note:* This table classifies the strength of evidence linking each therapeutic class to AMPK‐associated mechanisms and summarises their dominant downstream metabolic effects. Evidence strength is categorised based on the consistency of data across human randomised controlled trials (RCTs), ex vivo human tissues and rodent models.

Abbreviations: AMPK, AMP‐activated protein kinase; DNL, de novo lipogenesis; eNOS, endothelial nitric oxide synthase; FAO, fatty acid oxidation; GLP‐1 RA, glucagon‐like peptide‐1 receptor agonist; HIF‐1⍺, hypoxia‐inducible factor‐1 alpha; LKB1, liver kinase B1; PPAR𝛾, peroxisome proliferator‐activated receptor‐gamma; RCT, randomised controlled trial; ROCK, Rho‐associated protein kinase; SGLT2, sodium‐glucose cotransporter‐2; THR‐β, thyroid hormone receptor‐beta.

^a^ Direct hepatic signalling (e.g., LKB1‐AMPK activation) for SGLT2 inhibitors and GLP‐1 receptor agonists has been consistently observed in rodent models; however, evidence in human hepatocytes remains limited or suggests predominantly systemic mediation.

## 2 Pioglitazone: The Established PPARγ Agonist Engaging the Hepatic AMPK Axis

Pioglitazone is presently the most thoroughly investigated pharmacotherapy for MASH and functions as a standard for metabolic efficacy. It was originally developed for the treatment of type 2 diabetes [30], but it has been shown to confer histological benefits [31]. It is a strong agonist of the peroxisome proliferator‐activated receptor‐gamma (PPARγ) [32], which is a nuclear transcription factor that is predominantly expressed in adipose tissue [33]. Pioglitazone significantly enhances systemic insulin sensitivity [34] and diminishes the influx of free fatty acids to the liver by facilitating adipocyte differentiation and lipid storage in subcutaneous rather than visceral depots [35].

**FIGURE 1:**
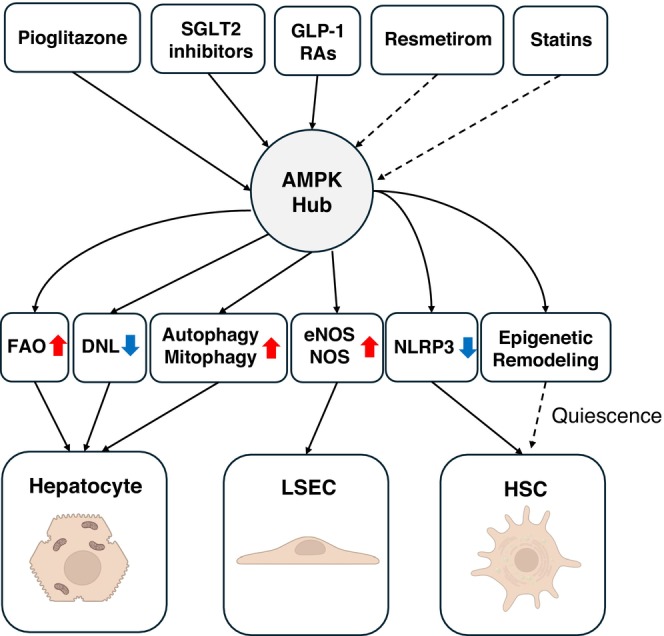
Diverse upstream cues converge on an AMPK hub to produce shared antifibrotic outcomes in MASH. SGLT2 inhibitors induce an energy‐deficit state that activates LKB1–AMPK; GLP‐1 receptor agonists act predominantly via systemic weight loss and insulin‐sensitization (with context‐limited CaMKKβ–AMPK); the THR‐β agonist resmetirom accelerates hepatocellular metabolism, shifting energy charge (±AMPK engagement); statins reduce isoprenoid‐dependent RhoA/ROCK signalling and trigger cellular‐stress–LKB1–AMPK while attenuating HIF‐1α‐driven programs. Downstream, AMPK restores microvascular function (eNOS/NO), suppresses NLRP3, enhances autophagy/mitophagy, decreases de novo lipogenesis and promotes HSC quiescence. Solid lines = established; dashed = context‐dependent/hypothesized. DNL, de novo lipogenesis; FAO, fatty‐acid oxidation; HSC, hepatic stellate cell; LSEC, liver sinusoidal endothelial cell.

Pioglitazone seamlessly integrates into the AMPK‐centric framework delineated in this review (Figure 1), primarily via an adipose‐liver endocrine axis. PPARγ activation promotes the release of adiponectin, an adipokine that is significantly inhibited in obesity and MASH [36]. Adiponectin in the blood binds to AdipoR1 and AdipoR2 receptors on the surface of hepatocytes [37]. This procedure brings in the adaptor protein APPL1, which turns on liver kinase B1 (LKB1). LKB1 then phosphorylates AMPK at Thr‐172 [38], which turns on the kinase. This ‘indirect’ activation occurs due to adipokine signalling rather than direct drug uptake by hepatocytes, leading to the usual AMPK effects: inhibition of acetyl‐CoA carboxylase (ACC), lowering de novo lipogenesis and enhancement of mitochondrial fatty acid oxidation [39].

Clinical evidence strongly supports this mechanism. Landmark randomised controlled trials, including the PIVENS [17] trial and the Belfort et al. study [6] have demonstrated significant improvements in steatosis, lobular inflammation and hepatocyte ballooning. Studies have even demonstrated the potential for fibrosis to regression [40, 41]. Elevated plasma adiponectin levels significantly correlate with these histological enhancements [42], confirming the adiponectin‐AMPK axis as the primary mediator of its therapeutic efficacy.

The clinical utility of pioglitazone is limited by its safety profile, which encompasses dose‐dependent weight gain, fluid retention, an elevated risk of bone fractures in postmenopausal women and potential concerns related to bladder cancer. These limitations highlight the intrinsic ‘trade‐off’ in first‐generation metabolic therapies: while pioglitazone effectively activates the AMPK axis to ameliorate liver damage, its systemic side effects often hinder long‐term adherence. This situation calls attention to the newer types of drugs that try to restore liver metabolism in a similar way but with better safety profiles.

## 3 SGLT2 Inhibitors: Systemic Metabolic Effects With Indirect Hepatic Benefits

Sodium–glucose cotransporter‐2 (SGLT2) inhibitors, such as empagliflozin and dapagliflozin, are antidiabetic medications that have demonstrated efficacy in MASLD/MASH [43, 44, 45]. They cause glycosuria by stopping the kidneys from reabsorbing glucose. This process lowers blood glucose and insulin levels, encourages mild osmotic diuresis and helps people lose weight. The liver, while not a direct target (hepatocytes exhibit minimal baseline SGLT2 expression), benefits from the enhanced systemic metabolic environment. SGLT2 inhibitors induce a physiological state comparable to caloric restriction or fasting, mitigating the factors that promote hepatic fat accumulation [46, 47]. Indeed, these agents have demonstrated reductions in hepatic steatosis, inflammation and fibrosis in preclinical models through multiple mechanisms [48, 49]: enhanced fatty acid oxidation, reduced de novo lipogenesis, decreased oxidative stress and modulation of inflammatory pathways [47, 50]. SGLT2 inhibitors have been shown to improve liver enzyme levels [51], lower liver fat levels on imaging [51, 52] and even improve fibrosis markers in patients.

**FIGURE 2:**
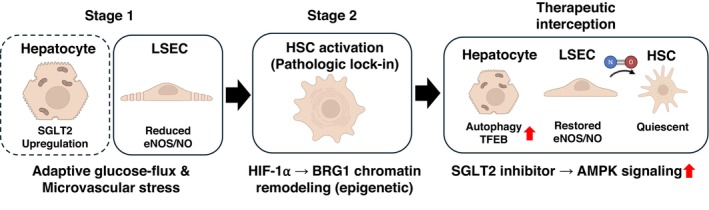
Two‐stage model of the hepatic SGLT2 axis. Stage 1: Hepatocyte SGLT2 upregulation (context‐dependent; dashed) and LSEC reduced eNOS/NO (solid) arise under metabolic/inflammatory stress, reflecting adaptive glucose‐flux control and microvascular dysfunction. Stage 2: Persistent injury drives HSC activation through HIF‐1α‐mediated chromatin remodelling via BRG1, leading to epigenetic lock‐in. Therapeutic interception: Pharmacologic SGLT2 inhibition induces an energy‐deficit → AMPK signal. Downstream effects are separated by cell type—LSEC: eNOS/NO restored and Hepatocyte: Autophagy/TFEB↑—with paracrine NO promoting HSC quiescence. Solid elements indicate relationships supported in humans; dashed elements denote context‐limited or debated links.

The advantages in the liver are predominantly indirect in nature [53, 54]. The drop in insulin levels and the rise in insulin sensitivity stop lipogenesis in hepatocytes that is caused by too much insulin. SGLT2 inhibitors can help people lose weight, usually a few kilograms [55]. This step helps reduce visceral fat, which lowers the flow of free fatty acids to the liver. The induced glycosuria also causes an energy loss that raises the hepatic AMP/ATP ratios. This activates AMPK through the liver's energy sensor LKB1 [56, 57]. This activation of AMPK changes the metabolism of hepatocytes so that they break down things instead of making them [58]. The process speeds up autophagy and fat oxidation while slowing down lipogenic and fibrogenic pathways [59]. A recent study demonstrated that in a MASH mouse model, an SGLT2 inhibitor increased hepatic autophagic flux via AMPK–TFEB (transcription factor EB) activation, while simultaneously decreasing steatosis, inflammation, and fibrosis (Figure 2).

New information suggests that MASH may have some direct effects on the liver once it is established [60]. In healthy conditions, SGLT2 is minimally expressed in liver cells; however, in the context of MASH, hepatocyte SGLT2 expression has been reported to increase, possibly triggered by hyperglycemia and inflammation. In vitro studies demonstrated that the administration of an SGLT2 inhibitor to hepatocytes under elevated glucose and lipid conditions inhibited glucose uptake, thereby diminishing pathological O‐GlcNAcylation (a signal of nutrient excess) and reactivating autophagy, which ultimately mitigated inflammation [20]. Additionally, SGLT2 inhibitors may affect non‐parenchymal cells; a murine study indicated that these drugs directly alter liver macrophages, transitioning them from a pro‐inflammatory (M1) to an anti‐inflammatory (M2) phenotype, irrespective of glycemic variations [61]. These findings indicate that even in a tissue that initially does not express SGLT2, the pathological state can establish sites of interaction for the drug.

In conclusion, SGLT2 inhibitors enhance hepatic outcomes primarily through systemic metabolic modulation, characterised by a sequence of reduced glucose and insulin levels, caloric deficit, weight loss and increased insulin sensitivity [62, 63], which collectively alleviate the factors contributing to steatotic and inflammatory liver injury. The innovative mechanism here is using a renal‐targeted mechanism to treat a liver disease indirectly. Although the direct hepatic effects of SGLT2 inhibitors are still being studied, current evidence strongly supports their use as metabolic therapy for MASH. Numerous clinical trials and meta‐analyses now validate that SGLT2 inhibitors can diminish liver fat and inhibit fibrosis progression in patients with MASLD [44, 64, 65, 66], rendering them a compelling choice, particularly for MASH patients with concurrent type 2 diabetes.

## 4 GLP‐1 Receptor Agonists

### 4.1 Hormonal Modulation With Multi‐Faceted Liver Impact

Glucagon‐like peptide‐1 receptor agonists (GLP‐1 RAs), including liraglutide and semaglutide, were originally formulated for type 2 diabetes and obesity [67]; however, they have shown significant effectiveness in MASH [11]. Semaglutide has demonstrated efficacy in resolving steatohepatitis in a considerable percentage of patients [11]. The primary mechanism of GLP‐1 RAs is by affecting the whole body [68]. They increase insulin secretion that depends on glucose, stop glucagon release and most importantly, they lower appetite, which can lead to a lot of weight loss (often 10%–15% of body weight with high‐dose semaglutide) [69]. This weight loss and better insulin sensitivity take some of the stress off the liver's metabolism. Caloric intake decreases, and adipose tissue mass diminishes, leading to reduced ectopic fat accumulation in hepatocytes and enhanced adipokine profiles. In fact, significant weight loss has been associated with substantial decreases in liver fat (up to approximately 81% relative reduction in liver fat content in certain studies of diet‐induced weight loss) [70], and GLP‐1 receptor agonists utilise this principle pharmacologically [71].

A major topic of conversation has been whether GLP‐1 RAs have direct effects on the liver as well. GLP‐1 receptors are abundantly expressed in pancreatic beta cells and specific brain regions [72, 73]; however, their expression in human hepatocytes is minimal to undetectable [22, 74, 75]. Recent comprehensive studies demonstrate that direct hepatic GLP‐1 receptor signalling is negligible [76]. Recent experiments on primary human hepatocytes and hepatic stellate cells using GLP‐1 and GIP agonists revealed no direct effects; the drugs failed to stimulate intracellular signalling or diminish fat accumulation and fibrogenesis in these cells [22]. This study indicates that the therapeutic advantages of GLP‐1 receptor agonists in metabolic‐associated steatotic hepatitis (MASH) are predominantly indirect, facilitated by enhancements in systemic metabolism—specifically, weight reduction, glycemic regulation and insulin sensitization—which subsequently foster a hepatic environment favourable for recovery (decreased lipogenesis, improved fat utilisation and diminished inflammation).

However, the literature is not completely biased. There have been indications of direct hepatic effects of GLP‐1, especially in rodent models [77]. Some research has identified GLP‐1 receptors on rat or mouse hepatocytes or on non‐parenchymal cells and has documented GLP‐1–induced activation of AMPK in hepatocytes or a decrease in steatosis in hepatocyte cultures. One suggested mechanism posits that the presence of GLP‐1 receptors on hepatocytes or stellate cells could activate a Ca^2+^‐dependent pathway through CaMKKβ, resulting in AMPK activation and thereby directly affecting lipid metabolism or fibrogenesis. Nevertheless, these results have not consistently been applicable to human physiology [76, 78]. It is still possible that hepatic GLP‐1R expression is increased in some pathological or developmental situations, or that GLP‐1 RAs have direct effects on cells like Kupffer cells or liver sinusoidal endothelial cells, which then have indirect effects on hepatocytes. The prevailing consensus indicates that direct hepatic GLP‐1 receptor agonist effects are constrained, with the majority of benefits arising from systemic effects [28].

The effect of GLP‐1 RAs on MASH has been very noticeable in the clinic. In the Phase 3 ESSENCE trial, weekly semaglutide 2.4 mg resulted in NASH resolution (without exacerbating fibrosis) in 62.9% of patients, compared to approximately 20% on placebo. Additionally, it achieved at least a one‐stage improvement in fibrosis in 36.8% of patients, versus 22.4% on placebo [11]. This level of effectiveness is comparable to that observed with intensive lifestyle interventions and highlights the importance of addressing the underlying causes of MASH (overeating and insulin resistance). It is significant that the improvement in fibrosis in the semaglutide trial, although considerable, was less pronounced than the NASH resolution rate. The difference indicates that reversing fibrosis may necessitate prolonged treatment or combinatorial strategies, potentially due to the inherent inertia of fibrosis [79] or the previously mentioned epigenetic memory in stellate cells [80].

From a therapeutic positioning perspective, GLP‐1 receptor agonists such as semaglutide are particularly advantageous for patients with MASH who also suffer from obesity or type 2 diabetes, which is a frequent occurrence. They target several cardiometabolic risk factors at once, providing holistic benefits (weight loss, better glycemic control, lower risk of cardiovascular events) that go beyond the liver [81]. The main problems are that it can cause stomach problems (nausea, vomiting and diarrhoea are common when the dose is increased) and that it is costly [11, 82]. Following these promising results, semaglutide received FDA approval for the treatment of MASH with moderate to advanced fibrosis in August 2025 [83].

In short, GLP‐1 RAs are a type of metabolic therapy for MASH that works on many levels. They strongly reset the energy balance and hormonal signals that cause fat to build up in the liver and inflammation. Their effect on the liver is mostly indirect, through weight loss and making insulin more sensitive, but eventually, many patients see a big improvement in liver histology. Ongoing research will further elucidate any minor direct pathways (e.g., portal‐nerve or immune cell‐mediated effects), but even in their current understanding, GLP‐1 RAs represent a cornerstone of MASH therapy by virtue of treating the metabolic syndrome at the heart of the disease [83].

### 4.2 Controversy and Exceptions: Do GLP‐1 RAs Act Directly on the Liver?

Our main interpretation is that GLP‐1 RAs enhance MASH mainly through systemic effects, such as weight loss, insulin sensitization and decreased nutrient flow to the liver. Nevertheless, reports concerning direct hepatic actions are inconsistent [78], exhibiting species‐, cell type‐ and disease state–specific variations that necessitate a judicious evaluation.

Evidence opposing substantial direct actions (human‐centric). Research utilising primary human hepatocytes and hepatic stellate cells often demonstrates negligible or absent GLP‐1R signalling, along with no direct diminution in steatosis or fibrogenic indicators under regulated conditions [22]. Human liver single‐cell atlases typically indicate low or undetectable GLP‐1R transcripts in parenchymal cells, implying that the majority of clinical benefits are derived indirectly through systemic metabolic enhancement.

Evidence for context‐restricted direct and parahepatic actions. In rodent models, hepatocyte or stellate‐cell GLP‐1R expression has been documented, accompanied by reports of AMPK activation and lipid reduction following GLP‐1RA exposure [84]. Certain investigations involving non‐parenchymal human cells (e.g., Kupffer cells, endothelial cells) or neuro‐hepatic circuits suggest para‐hepatic mechanisms that secondarily influence hepatocytes [85]. Disease‐state remodelling may also be significant: pathologic upregulation of GLP‐1R in injured liver or indirect receptor‐independent peptide fragments (e.g., short GLP‐1 metabolites with mitochondrial effects) have been suggested [76], although their translational relevance remains limited thus far.

The process of synthesis is currently underway. In humans, the primary focus remains on indirect systemic mediation, with direct or para‐hepatic signals likely occurring only in specific scenarios such as species differences, non‐parenchymal targets, or disease‐induced changes in receptors. We propose that direct hepatic actions may be possible but are not the primary drivers. Additionally, we emphasise testable predictions (e.g., receptor localization in diseased human liver, cell‐type–resolved phospho‐signalling and peptide‐fragment pharmacology) to clarify the debate.

## 5 Thyroid Hormone Receptor‐β Agonist (Resmetirom): Liver‐Targeted Metabolic Activation

Resmetirom (MGL‐3196) is a selective thyroid hormone receptor‐β agonist that is meant to selectively activate thyroid hormone signalling in the liver. Thyroid hormone is a key regulator of basal metabolic rate and lipid metabolism [86]. Resmetirom aims to obtain the metabolic benefits (more fat burning and lower cholesterol) without the systemic side effects of too much thyroid hormone (like heart stimulation) by only targeting the β isoform of the thyroid hormone receptor (the most common form in the liver) [23].

Resmetirom works in hepatocytes by turning up the expression of genes that are involved in mitochondrial β‐oxidation and energy use and turning down the expression of genes that are involved in making fat and glucose [87]. In essence, it brings about a condition akin to mild hyperthyroidism localised to the liver [88]. This results in a decrease in hepatic triglyceride levels (through enhanced fatty acid oxidation and export) [89] and amelioration of dyslipidemia (resmetirom enhances LDL receptor expression, facilitating more effective clearance of LDL cholesterol). Resmetirom can lower the extra fat and toxic lipid intermediates that cause steatohepatitis by speeding up metabolism in hepatocytes. There is also evidence that thyroid hormone signalling can directly decrease fibrosis and inflammation, potentially via interactions with hepatic stellate cell activity and macrophage polarisation [90], although these effects are less thoroughly defined than the metabolic ones.

Resmetirom has demonstrated substantial efficacy in clinical settings for MASH [24]. In the Phase 3 MAESTRO‐NASH trial, resmetirom (80 mg and 100 mg) achieved NASH resolution in 25.9% and 29.9% of patients, respectively, compared to 9.7% with placebo (*p* < 0.001). Furthermore, fibrosis improvement by at least one stage occurred in 24.2% and 25.9% of the treatment groups, versus 14.2% in the placebo group (*p* < 0.001) [24]. The FDA approved resmetirom because of these results, along with big drops in liver fat on MRI‐PDFF and better liver enzymes. Resmetirom had a positive effect on fibrosis, but it wasn't forceful. Its main strength was in getting rid of steatosis and inflammation, which set the stage for fibrosis to go away over time [91]. This profile goes well with drugs that might have more direct effects on fibrotic tissue.

Resmetirom is a liver‐centric engine booster that directly speeds up liver metabolism [92]. A rational strategy involves combining it with drugs like GLP‐1 RAs or SGLT2 inhibitors that work more on systemic factors, which would attack MASH from both inside and outside the liver. Overall, the safety profile of resmetirom has been satisfactory [9]. Because thyroid hormone can speed up the heart rate and cause arrhythmias [93], careful dose titration was used in trials. At the approved doses, there were only small increases in resting heart rate, and thyroid function tests stayed within the normal range for most patients. Some patients have temporary hyperthyroid‐like symptoms, such as jitteriness or mild increases in liver enzymes. However, serious side effects have been rare. Long‐term safety is still being looked into [94], especially since the pill is a long‐term treatment.

Resmetirom is also related to the common theme of AMPK activation, but not directly [25]. Thyroid hormone can lower the energy charge of hepatocytes by accelerating processes that use ATP, such as increasing the activity of Na^+^/K^+^ ATPase and other metabolic cycles in the liver [95]. The increased activity may then activate AMPK. Thyroid hormone signalling also interacts with pathways such as fibroblast growth factor 21 (FGF21) production, which has positive effects on metabolism [96, 97]. Therefore, resmetirom can be seen as both taking away the ‘fuel substrate’ (fat) and possibly starting stress pathways that help the liver.

In conclusion, resmetirom presents an innovative mechanism of action within the MASH therapeutic framework: targeted hormonal enhancement of hepatic metabolism. Its approval marks a pivotal moment in the field, demonstrating that targeting essential metabolic pathways in the liver can effectively treat the disease. Resmetirom is the first drug that works specifically for MASH [88, 98]. It sets a standard for future treatments, and it will probably be used with other drugs (e.g., with a GLP‐1 RA for an obese MASH patient to help with both weight and liver, or with an SGLT2 inhibitor for a diabetic MASH patient to help with blood sugar and liver control).

## 6 Statins: Pleiotropic Metabolic Benefits and Stress Signalling Modulation

Statins, also known as hydroxymethylglutaryl‐CoA (HMG‐CoA) reductase inhibitors, are commonly used to treat dyslipidemia and lower the risk of heart disease [99]. Statins were not created for MASH, but they do have important effects that are related to MASH pathophysiology. Epidemiological studies encompassing extensive liver disease populations have identified a correlation between statin use and a diminished risk of hepatic decompensation and hepatocellular carcinoma [100]. In individuals with MASLD or MASH, statins appear to enhance serum liver enzymes and may decelerate fibrosis progression [101, 102]—probably as a secondary advantage of their cardiovascular risk mitigation and possibly through direct effects on liver cells.

The primary mechanism of statins is the inhibition of the mevalonate pathway in the liver, which lowers cholesterol production. This increases LDL receptors and speeds up LDL removal from the blood (which is good for the heart), but it also affects MASH cells. Statins can change how cells talk to each other by lowering the levels of cholesterol and isoprenoid intermediates inside cells. One well‐known effect is that it stops the small GTPase Rho and its downstream effector Rho‐kinase (ROCK) from working [103]. This fact is because Rho needs geranylgeranylation (a process that depends on mevalonate) to work [104]. In hepatic cells, RhoA/ROCK activity plays a role in inflammatory and fibrogenic responses [105]. Statins, by inhibiting RhoA/ROCK signalling, have demonstrated the capacity to decrease pro‐inflammatory cytokine production and may indirectly reduce the activation of hepatic stellate cells [106]. Experimental models of MASH have shown that statins can stop stellate cells from becoming active and even help fibrosis go away by increasing endothelial nitric oxide synthase and improving microvascular function [26, 107].

Statin therapy also causes a small amount of stress in the endoplasmic reticulum and mitochondria of hepatocytes [108]. Cholesterol is necessary for cell membranes and mitochondrial function; coenzyme Q10, an essential part of the electron transport chain, is a product of the mevalonate pathway [109]. Using statins to lower cholesterol and coenzyme Q10 can lead to energy strain. The liver cell may interpret the condition as a low‐energy state, subsequently activating LKB1‐AMPK as a compensatory mechanism. In fact, there have been reports that statins can turn on AMPK in some situations, which helps to stop lipids from building up [27]. Additionally, by decreasing Rho/ROCK activity, statins affect the hypoxia‐inducible factor‐1α (HIF‐1α) pathway [110]. HIF‐1α is frequently upregulated in MASH livers, attributable to regions of relative hypoxia in fatty liver and an inflammatory environment, thereby facilitating fibrogenic gene expression [111, 112, 113]. Rho/ROCK signalling can increase the transcriptional activity of HIF‐1α [114]. On the other hand, statin‐induced Rho inhibition has been associated with lower levels and activity of HIF‐1α [115]. The overall result is that HIF‐1α target genes like collagen and plasminogen activator inhibitor‐1 are expressed less, which may slow down fibrosis [116].

Statins have more than just molecular effects. They also have general anti‐inflammatory effects, like lowering CRP and changing how immune cells are recruited, and they improve endothelial function in the liver microcirculation [117]. These effects help break the cycle of injury in MASH [118]. Enhanced endothelial nitric oxide signalling can diminish hepatic vascular resistance and portal pressure, subsequently improving sinusoidal perfusion and alleviating tissue hypoxia, thereby indirectly benefiting hepatocytes and stellate cells [26, 119].

It is important to note that statins are not a specific treatment for NASH [120]. But since MASH patients often have metabolic syndrome, a lot of them already need statins. The synergy is useful: treating cardiovascular risk may help protect the liver. In a systematic review and real‐world analyses, statin therapy in MASLD/MASH cohorts has been correlated with reduced progression to advanced fibrosis and a tendency towards improvement in fibrosis scores [121]. There was no rise in hepatotoxicity in these patients beyond the baseline low risk associated with statins [122], confirming that statins are safe for individuals with liver disease and should not be avoided due to unfounded concerns about hepatotoxicity in MASLD [123]. On the other hand, a MASH patient with high LDL or a high risk of heart disease should be on a statin unless there is a reason not to [124]. Their use could have two benefits.

In summary, statins are used to lower cholesterol to prevent heart disease, but they also have other effects that are advantageous for MASH, such as blocking Rho/ROCK and affecting the AMPK and HIF‐1α pathways. They lessen the amount of substrate (cholesterol and maybe other lipotoxic sterols) that can make liver damage worse, calm down inflammatory and fibrogenic signalling and make the liver's microenvironment better. Adding statins to a MASH patient's treatment plan takes care of the linked risk of heart disease and worsening liver fibrosis. Future trials may further quantify statins' antifibrotic potential in MASH, but even now, their use exemplifies how addressing systemic metabolic risk factors can positively influence liver outcomes.

## 7 The AMPK‐Epigenetic Axis: A Convergent Downstream Consequence of Metabolic Restoration

**FIGURE 3:**
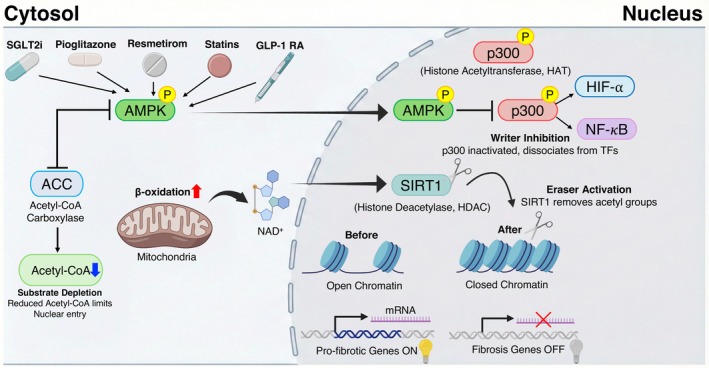
The AMPK‐epigenetic axis. Proposed mechanism by which metabolism‐based therapies converge on AMPK to induce transcriptional repression of pro‐fibrotic genes in hepatic stellate cells. The diagram illustrates three synergistic pathways driving chromatin remodelling: (1) substrate depletion via ACC inhibition (limiting acetyl‐CoA); (2) writer inhibition via p300 phosphorylation; and (3) eraser activation via the NAD^+^‐SIRT1 axis. Collectively, these actions promote a transition from an open, active chromatin state to a closed, repressive state (Quiescence). ACC, acetyl‐CoA carboxylase; AMPK, AMP‐activated protein kinase; HAT, histone acetyltransferase; HDAC, histone deacetylase; HIF‐1⍺, hypoxia‐inducible factor‐1 alpha; HSC, hepatic stellate cell; NAD^+^, nicotinamide adenine dinucleotide; NF‐𝜅B, nuclear factor kappa B; SIRT1, Sirtuin 1.

One of the most perplexing challenges in MASH treatment is ‘therapeutic inertia’, defined as the persistence or progression of fibrosis despite metabolic improvement. We and others propose that this phenomenon is driven by ‘epigenetic lock‐in’, a state where chronic metabolic stress (lipotoxicity, hyperglycemia) induces stable chromatin modifications in hepatic stellate cells (HSCs) and hepatocytes. This process sustains a pathological gene expression program even after the cessation of the initial insult. Metabolism‐based therapies (SGLT2 inhibitors, GLP‐1 RAs, pioglitazone, resmetirom and statins) primarily target systemic and hepatic energy homeostasis. We hypothesize that their durable efficacy is contingent upon their ability to reverse this epigenetic state. In this convergent model, we present AMPK activation as a metabolic‐epigenetic transducer, connecting energy sensing directly to chromatin remodelling through three distinct molecular pathways (Figure 3; Table 2).

**TABLE 2:** Proposed AMPK‐mediated epigenetic modifications in MASH fibrosis.

Epigenetic target	Role in MASH fibrosis	AMPK‐mediated mechanism	Therapeutic outcome
Acetyl‐CoA	Substrate for HATs	Substrate depletion Phosphorylation of ACC lowers cytosolic acetyl‐CoA pool	‘Starving’ the epigenome Reduces global histone acetylation at pro‐fibrotic loci
p300/CBP	HATs that activate pro‐inflammatory genes	Direct inhibition Direct phosphorylation at Ser89 inhibits HAT activity	Transcriptional repression Disrupts p300‐HIF‐1⍺ and NF‐𝜅B complex formation
SIRT1	HDAC; Anti‐inflammatory	NAD^+^ activation Increased mitochondrial oxidation raises NAD^+^ levels	Chromatin compaction Promotes deacetylation and silencing of inflammatory genes
BRG1	Chromatin Remodeler (SWI/SNF complex)	Indirect downregulation Resolution of metabolic stress reduces BRG1 recruitment	Reversing ‘Lock‐in’ Prevents maintenance of HSC activation memory

*Note:* Summary of how AMPK activation by metabolism‐based therapies can transduce metabolic signals into chromatin remodelling, potentially reversing the ‘epigenetic lock‐in’ of hepatic stellate cells.

Abbreviations: ACC, acetyl‐CoA carboxylase; AMPK, AMP‐activated protein kinase; BRG1, Brahma‐related gene 1; CBP, CREB‐binding protein; HAT, histone acetyltransferase; HDAC, histone deacetylase; HIF‐1α, hypoxia‐inducible factor‐1 alpha; HSC, hepatic stellate cell; NAD^+^, nicotinamide adenine dinucleotide; NF‐κB, nuclear factor kappa B; SIRT1, Sirtuin 1.

### 7.1 Starving the Epigenome (Acetyl‐CoA Depletion)

Histone acetylation, which generally opens chromatin to facilitate pro‐fibrotic gene expression, relies entirely on the availability of the donor substrate, acetyl‐CoA. In the nutrient‐rich environment of MASH, high cytosolic acetyl‐CoA levels drive the ‘opening’ of fibrogenic loci. We propose that therapeutic activation of AMPK reverses this process by phosphorylating and inhibiting acetyl‐CoA carboxylase (ACC). This inhibition reduces de novo lipogenesis and restricts the cytosolic pool of acetyl‐CoA available for histone acetyltransferases (HATs). AMPK activation creates a localised ‘substrate scarcity’ that effectively starves the epigenetic machinery. This process reduces the acetylation of histone H3 lysine 27 (H3K27ac) at the promoters of pro‐inflammatory and pro‐fibrotic genes, such as *COL1A1* and *ACTA2*. This mechanism elucidates the molecular basis by which energy‐deficit‐mimicking drugs (SGLT2 inhibitors) or insulin sensitizers (pioglitazone) can suppress activated HSCs.

### 7.2 Direct Inhibition of Chromatin Writers (p300/CBP)

Beyond substrate control, AMPK directly regulates chromatin ‘writers’. The transcriptional co‐activator p300/CBP is a potent histone acetyltransferase known to stabilise HIF‐1⍺ and NF‐𝜅B, key drivers of inflammation and fibrosis in MASH. AMPK directly phosphorylates p300 at Serine 89, which inhibits its transcriptional activity. In the context of MASH, drug‐induced AMPK activation would consequently disrupt the p300‐HIF‐1⍺ complex. This effect is particularly relevant for statins and SGLT2 inhibitors, which have been linked to reduced HIF‐1⍺ signalling. By disrupting this interaction, AMPK facilitates the transition of chromatin from an active, inflammatory state to a repressive, quiescent state.

### 7.3 The AMPK‐SIRT1 Deacetylation Axis (Eraser Activation)

Finally, AMPK works in concert with Sirtuin 1 (SIRT1), a Class III histone deacetylase that functions as a key chromatin eraser dependent on NAD+. AMPK elevates intracellular NAD^+^ levels by accelerating mitochondrial fatty acid oxidation and regulating the NAMPT salvage pathway. The resulting rise in the NAD^+^/NADH ratio activates SIRT1, which removes acetyl groups from histones and non‐histone proteins, such as NF‐κB and FOXO1. This AMPK‐SIRT1 axis creates a powerful ‘double‐hit’ against fibrosis: AMPK prevents the addition of acetyl groups (by inhibiting p300 and depleting acetyl‐CoA), while SIRT1 promotes their removal.

### 7.4 From Theory to Validation

The concept of epigenetic lock‐in explains the ‘metabolic memory’ observed in HSCs, where cells remain primed for fibrogenesis due to persistent chromatin remodelling mediated by factors such as BRG1 (Brahma‐related gene 1). Current evidence indicates that although metabolic enhancement is essential, it may be inadequate without profound cellular reprogramming. To clinically substantiate this AMPK‐epigenetic association, subsequent trials must transcend conventional histology. We suggest the integration of epigenomic profiling (e.g., ChIP‐seq for H3K27ac, ATAC‐seq for chromatin accessibility) on paired liver biopsies obtained prior to and following treatment with AMPK‐activating agents. Demonstrating that a pharmacological regimen (e.g., Pioglitazone + GLP‐1 RA) induces a ‘closed’ chromatin signature at fibrotic loci would constitute conclusive evidence for the potential to reverse epigenetic lock‐in.

## 8 Conclusions

The therapeutic landscape for MASH is transitioning from a dependence on lifestyle modification to a diverse spectrum of metabolism‐based pharmacotherapies. In this review, we proposed a mechanistic framework centred on AMPK as a convergent signalling node, rather than a singular uniform target. We illustrated how distinct upstream triggers—spanning the established PPARγ‐adiponectin axis of pioglitazone and the thyroid hormone receptor‐mediated mitochondrial turnover of resmetirom to the systemic energy deficits induced by SGLT2 inhibitors and GLP‐1 receptor agonists—converge into shared downstream pathways that restore hepatic energy homeostasis and alleviate cellular stress.

Our analysis underscores that while the degree of direct hepatic engagement varies among these agents, their collective efficacy depends on overcoming ‘epigenetic lock‐in’. We posit that the durability of MASH treatment lies in the ability of these metabolic interventions to transduce signals to the chromatin level—specifically by depleting the acetyl‐CoA pool, inhibiting HATs like p300 and activating the SIRT1 axis—thereby erasing the ‘metabolic memory’ of hepatic stellate cells.

Moving forward, the integration of these agents into combination therapies offers a rational strategy to target complementary pathogenic drivers. However, the translation of these mechanistic synergies into clinical practice requires vigilance regarding overlapping safety profiles (e.g., gastrointestinal tolerance, volume status) and distinct adverse events. Future research should prioritise validating the AMPK‐epigenetic axis in human biopsies and identifying non‐invasive biomarkers that reflect these chromatin states. Ultimately, this convergent molecular perspective provides a foundation for precision medicine, shifting the goal from mere metabolic management to the fundamental cellular reprogramming of the diseased liver.

## Author Contributions

Conceptualization: Seungchan Choi, Jin‐Seok Jung, Kangchan Choi; data curation: Kangchan Choi; investigation: Seungchan Choi, Jin‐Seok Jung, Yie‐sung Seo, Sungmin Song, Jeehye Ham, Hannah Chung, Yousef Ramadan, Kangchan Choi; project administration: Kangchan Choi; resources: Seungchan Choi, Jin‐Seok Jung, Kangchan Choi; supervision: Kangchan Choi; visualisation: Kangchan Choi; writing – original draft: Seungchan Choi, Jin‐Seok Jung, Kangchan Choi; writing – review and editing: Seungchan Choi, Jin‐Seok Jung, Yie‐sung Seo, Sungmin Song, Jeehye Ham, Hannah Chung, Yousef Ramadan, Kangchan Choi. All authors have read and agreed to the published version of the manuscript.

## Funding

The authors have nothing to report.

## Conflicts of Interest

The authors declare no conflicts of interest.

## Data Availability

The authors have nothing to report.
